# Development of a COVID-19 early risk assessment system based on multiple machine learning algorithms and routine blood tests: a real-world study

**DOI:** 10.3389/fimmu.2024.1430899

**Published:** 2024-09-30

**Authors:** Qiangqiang Qin, Qingxuan Li, Guiyin Zhu, Haiyang Yu, Mingyan Peng, Shuang Wu, Xue Xu, Wen Gu, Xuejun Guo

**Affiliations:** ^1^ Department of Respiratory Medicine, Xinhua Hospital, Shanghai Jiaotong University School of Medicine, Shanghai, China; ^2^ Department of Respiratory and Critical Care Medicine, The Second Hospital of Jilin University, Changchun, Jilin, China; ^3^ Department of Gynecology and Obstetrics, Xinhua Hospital, Shanghai Jiaotong University School of Medicine, Shanghai, China; ^4^ Stomatologic Hospital and College, Anhui Medical University, Hefei, China

**Keywords:** COVID-19, machine learning, predictive model, categorized treatment, predictive preventive personalized medicine

## Abstract

**Backgrounds:**

During the Coronavirus Disease 2019 (COVID-19) epidemic, the massive spread of the disease has placed an enormous burden on the world’s healthcare and economy. The early risk assessment system based on a variety of machine learning (ML) algorithms may be able to provide more accurate advice on the classification of COVID-19 patients, offering predictive, preventive, and personalized medicine (PPPM) solutions in the future.

**Methods:**

In this retrospective study, we divided a portion of the data into training and validation cohorts in a 7:3 ratio and established a model based on a combination of two ML algorithms first. Then, we used another portion of the data as an independent testing cohort to determine the most accurate and stable model and compared it with other scoring systems. Finally, patients were categorized according to risk scores and then the correlation between their clinical data and risk scores was studied.

**Results:**

The elderly accounted for the majority of hospitalized patients with COVID-19. The C-index of the model constructed by combining the stepcox[both] and survivalSVM algorithms was 0.840 in the training cohort and 0.815 in the validation cohort, which was calculated to have the highest C-index in the testing cohort compared to the other 119 ML model combinations. Compared with current scoring systems, including the CURB-65 and several reported prognosis models previously, our model had the highest AUC value of 0.778, representing an even higher predictive performance. In addition, the model’s AUC values for specific time intervals, including days 7,14 and 28, demonstrate excellent predictive performance. Most importantly, we stratified patients according to the model’s risk score and demonstrated a difference in survival status between the high-risk, median-risk, and low-risk groups, which means a new and stable risk assessment system was built. Finally, we found that COVID-19 patients with a history of cerebral infarction had a significantly higher risk of death.

**Conclusion:**

This novel risk assessment system is highly accurate in predicting the prognosis of patients with COVID-19, especially elderly patients with COVID-19, and can be well applied within the PPPM framework. Our ML model facilitates stratified patient management, meanwhile promoting the optimal use of healthcare resources.

## 1 Introduction

Coronavirus Disease 2019 (COVID-19) is a disease caused by severe acute respiratory syndrome coronavirus type 2 (SARS-CoV-2), whose clinical manifestations commonly include fever, dry cough, and malaise. According to the World Health Organization, as of May 22, 2022, COVID-19 has caused more than 524 million infections and more than 6.27 million deaths (1). In addition to its high mortality rate, COVID-19 is highly contagious due to its multiple modes of transmission, rapid mutation and general susceptibility (2). COVID-19 has placed a significant economic burden on the global healthcare system, as the direct medical costs of symptomatic COVID-19 cases tend to be significantly higher than those of other common infectious diseases, and its rapid rate of transmission leads to a surge in public health management costs (3–5). Strategies such as vaccination or isolation to prevent and reduce transmission, stratified care, or individualized treatment to rationalize the use of medical resources have also been proposed to improve this chaos (4). How to optimize these strategies to achieve simpler and more accurate decision-making at the same time is a pressing issue.

An exploration of the clinical data of patients with COVID-19 from previous visits would be useful for this purpose. The enormous size and extreme complexity of clinical data have led researchers to increasingly prefer the use of artificial intelligence (AI)to deal with it, with machine learning (ML) as a subgroup of AI achieving notable success in building predictive models (6). ML, a scientific discipline that focuses on how computers learn from data, is applied to the diagnosis, prognosis, and treatment options for a variety of diseases (7, 8). To our knowledge, ML has been used in constructing prognostic models for COVID-19 patients, but most of them use only a single ML algorithm, which may lead to a high risk of bias (9). An integrated procedure based on various ML algorithms and their combinations may be a new approach to deep learning that can further reduce the dimensionality of variables and make models more simplified and transformative (10).

In order to provide a simpler and more refined classification of patients, our paper is the first to combine two ML algorithms based on blood tests to more accurately predict the prognosis of COVID-19 patients and to develop a risk scoring system based on the prognostic model for clinical use. In addition, we obtained good results by comparing the model with scoring currently used in clinical settings and other ML models. This suggests that the assessment system can accurately predict mortality in COVID-19 patients as quickly as possible, which could contribute to triage management of patients to reduce the burden of care on the one hand, and early identification of high-risk patients for immediate management to reduce mortality on the other.

## 2 Materials and methods

### 2.1 Data collection

We conducted a retrospective study including 282 patients who were admitted to Xinhua Hospital Affiliated to Shanghai Jiao Tong University School of Medicine between January 1, 2023 and March 1, 2023. Upon admission, we collected demographic data, baseline medical history, and laboratory test results for subsequent modeling and analysis. Our inclusion criteria consisted of three primary factors (1): age ≥18 years; (2) confirmation of COVID-19 infection by RT-PCR; and (3) availability of blood samples within 24 hours of admission. We excluded patients < 18 years and those with primary autoimmune or hematologic diseases to ensure that our study cohort was representative of the general population with COVID-19. After the application of our inclusion and exclusion criteria, a total of 265 eligible patients were enrolled. In addition, in order to avoid the over fitting of the model using the above data, we used the data set of 226 eligible COVID-19 patients collected from March 1, 2023 to March 1, 2024 as an independent cohort to determine the best model. Feature selection and model construction

To ensure the validity and accuracy of our modeling process, we divided 265 patients into training and validation cohorts with a random ratio of 7:3 (11), and the remaining 226 patients were set as an independent testing group. We list the baseline characteristics of the patients in the three cohorts and summarize the results of the laboratory tests on their blood. Variables with missing values exceeding 20% were deleted to preserve the majority of the original dataset. For variables with less than 20% of missing values, we employed appropriate imputation techniques to restore the missing data (12). In our study, we adopted 13 ML algorithms to generate a total of 119 ML model combinations (10, 13). We began by employing univariate Cox regression to screen for prognostic-related variables based on a significance threshold of P<0.05. To ensure optimal model stability, models with fewer than five variables were excluded. Lastly, we calculated the C-index for each combination algorithm in training, validation, and testing cohorts. The best model combination was chosen based solely on its performance in the testing cohort.

### 2.2 Collection and comparison of prognostic models for COVID-19

The CURB-65 score is a widely recognized and clinically useful system for assessing the prognosis of pneumonia patients and guiding treatment (14). To evaluate the predictive ability of our model, we utilized the “pROC” and “survivalROC” packages to calculate multi-variable ROC and time-dependent ROC curves in training, validation, and testing cohorts. With the increasing use of ML in guiding COVID-19 treatment and predicting prognosis, several models, have used liver function, coagulation, and hematological parameters to predict patient outcomes (15–17). To predict the outcomes of the patients in the training, validation, and testing cohorts, we utilized the C-index and variables obtained through machine learning, comparing them with the variables utilized in previously published literature. The results were then presented using a forest plot.

### 2.3 Model performance and analysis features incorporated in the model

To facilitate the allocation of clinical resources and disease risk stratification, the population was classified into three groups based on their mortality risks: high-risk group (top 15%), intermediate-risk group (middle 50%), and low-risk group (remaining population). By plotting Kaplan-Meier curves based on these groups, we were able to visualize the differences in survival rates. To further analyze the KM curves, we utilized the “pairwise_survdiff” function from the “survminer” package to compare the differences among the three curves. In this study, a series of clinical baseline data and laboratory examination indicators of patients were collected. We used the R software “ggcor” package to explore the correlation between risk score and demographic data, and laboratory test results, as well as the correlation between various variables, and the results were presented in the form of a butterfly graph. Moreover, to explore the correlation between risk score and underlying diseases, we compared the scores among patients, who had different underlying conditions.

### 2.4 Statistical analysis

Specifically, we compared the differences between groups for continuous variables using t-tests or Wilcoxon rank sum tests, while for categorical variables, we employed chi-squared tests. Moreover, we utilized the “survival” package for univariate Cox analysis and K-M curve drawing, and Pearson correlation was utilized to investigate the correlation between two continuous variables. We further plotted multivariable ROC curves and time-dependent ROC curves using the “pROC” and “survivalROC” packages, respectively. The DeLong test is employed to assess the discrepancy among ROC curves. All data statistical analyses were based on R 4.2.2, and unless otherwise stated, we determined P<0.05 to be statistically significant.

## 3 Results

### 3.1 Data acquisition and characteristics

**Figure 1:**
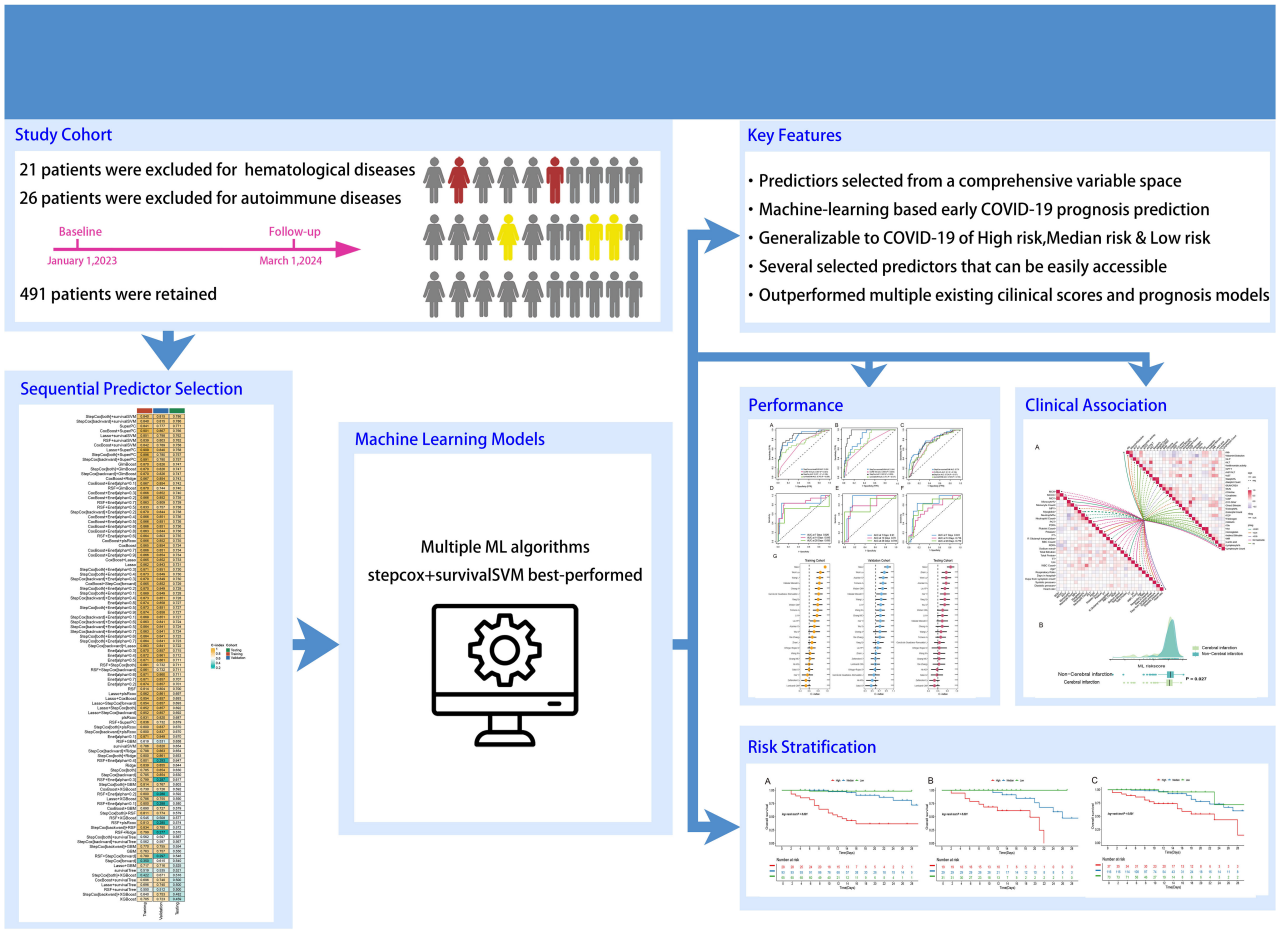
Flowchart of this research.

Initially, demographic data, baseline medical history, and laboratory test results were collected for a total of 534 individuals diagnosed with COVID-19. These data were meticulously compiled by three seasoned respiratory doctors. Thereafter, 15 patients with primary hematological diseases and 36 patients with autoimmune diseases were subsequently excluded from the analysis, leaving a final cohort of 491 COVID-19 patients and their corresponding data for subsequent analysis. A comprehensive summary of patients’ information can be found in **Table 1**. Out of the patients included in this study, 79 died (27 in the training cohort, 17 in the validation cohort, and 35 in the testing cohort) and 412 survived, with an average hospitalization duration of 11.00 [8.00;16.00] days, and duration from symptom onset to the admission of 10.00 [7.00;15.00] days. The prevalence of hypertension, diabetes, and coronary heart disease among the patients was 55.19%, 34.22%, and 27.7%, respectively, with the median age of the patients being 75. The findings align with previous studies (18–20). The study’s research process is visually presented in **Figure 1** as a comprehensive flowchart.

**Table 1:** Patients’ baseline characteristics in training, validation, and testing cohort.

	Training cohort (N=186)	Validation cohort (N=79)	Testing cohort (N=226)	Total (N=491)	P value
Gender (%)					0.834
- Female	82 (44.09)	32 (40.51)	100 (44.25)	214 (43.58)	
- Male	104 (55.91)	47 (59.49)	126 (55.75)	277 (56.42)	
Status (%)					0.345
- Alive	159 (85.48)	62 (78.48)	191 (84.51)	412 (83.91)	
- Dead	27 (14.52)	17 (21.52)	35 (15.49)	79 (16.09)	
supplement oxygen support (%)					0
- No	155 (83.33)	62 (78.48)	24 (10.62)	241 (49.08)	
- Yes	31 (16.67)	17 (21.52)	202 (89.38)	250 (50.92)	
Age (median [IQR])	74.00 [68.00;84.00]	78.00 [69.50;87.00]	72.00 [66.00;80.00]	74.00 [67.00;82.00]	0
RR (median [IQR])	19.00 [16.00;22.00]	19.00 [16.00;22.00]	20.00 [18.00;26.00]	19.00 [17.00;22.50]	0.021
Days in hospital (median [IQR])	13.00 [9.00;17.00]	13.00 [9.00;17.00]	10.00 [8.00;15.00]	11.00 [8.00;16.00]	0.006
Days from symptom onset (median [IQR])	11.00 [7.00;15.00]	11.00 [7.00;14.00]	10.00 [7.00;15.00]	10.00 [7.00;15.00]	0.399
Temperature (median [IQR])	36.70 [36.50;36.90]	36.70 [36.50;37.00]	36.60 [36.50;36.80]	36.60 [36.50;36.90]	0.056
Systolic pressure (mean (SD))	134.00 [120.00;150.00]	137.00 [126.00;150.00]	130.00 [120.00;142.00]	132.00 [120.50;146.50]	0.037
Diastolic pressure (mean (SD))	77.00 [68.00;86.00]	76.00 [71.00;86.50]	78.00 [71.00;85.00]	77.00 [70.00;85.00]	0.444
Heart rate (median [IQR])	88.00 [78.00;100.00]	90.00 [77.00;100.50]	84.00 [77.00;90.00]	86.00 [77.00;98.00]	0.018
COPD (%)					0
- No	170 (91.40)	69 (87.34)	222 (98.23)	461 (93.89)	
- Yes	16 (8.60)	10 (12.66)	4 (1.77)	30 (6.11)	
PE (%)					0.279
- No	171 (91.94)	75 (94.94)	216 (95.58)	462 (94.09)	
- Yes	15 (8.06)	4 (5.06)	10 (4.42)	29 (5.91)	
Lung cancer (%)					0.488
- No	176 (94.62)	75 (94.94)	219 (96.90)	470 (95.72)	
- Yes	10 (5.38)	4 (5.06)	7 (3.10)	21 (4.28)	
Hypertension (%)					0.007
- No	77 (41.40)	26 (32.91)	117 (51.77)	220 (44.81)	
- Yes	109 (58.60)	53 (67.09)	109 (48.23)	271 (55.19)	
Atrial fibrillation (%)					0
- No	151 (81.18)	65 (82.28)	219 (96.90)	435 (88.59)	
- Yes	35 (18.82)	14 (17.72)	7 (3.10)	56 (11.41)	
Coronary heart disease (%)					0.954
- No	135 (72.58)	56 (70.89)	164 (72.57)	355 (72.30)	
- Yes	51 (27.42)	23 (29.11)	62 (27.43)	136 (27.70)	
Cardiac insufficiency (%)					0.242
- No	154 (82.80)	65 (82.28)	199 (88.05)	418 (85.13)	
- Yes	32 (17.20)	14 (17.72)	27 (11.95)	73 (14.87)	
arrhythmia (%)					0.972
- No	161 (86.56)	69 (87.34)	195 (86.28)	425 (86.56)	
- Yes	25 (13.44)	10 (12.66)	31 (13.72)	66 (13.44)	
Endocrine diseases (%)					0
- No	108 (58.06)	35 (44.30)	165 (73.01)	308 (62.73)	
- Yes	78 (41.94)	44 (55.70)	61 (26.99)	183 (37.27)	
Diabetes (%)					0.001
- No	118 (63.44)	40 (50.63)	165 (73.01)	323 (65.78)	
- Yes	68 (36.56)	39 (49.37)	61 (26.99)	168 (34.22)	
Neurological disorders (%)					0.029
- No	132 (70.97)	64 (81.01)	184 (81.42)	380 (77.39)	
- Yes	54 (29.03)	15 (18.99)	42 (18.58)	111 (22.61)	
Parkinson disease (%)					0.094
- No	182 (97.85)	78 (98.73)	226 (100.00)	486 (98.98)	
- Yes	4 (2.15)	1 (1.27)	0 (0.0)	5 (1.02)	
Cerebral infarction (%)					0.268
- No	147 (79.03)	69 (87.34)	186 (82.30)	402 (81.87)	
- Yes	39 (20.97)	10 (12.66)	40 (17.70)	89 (18.13)	
History of malignancy (%)					0.117
- No	160 (86.02)	66 (83.54)	206 (91.15)	432 (87.98)	
- Yes	26 (13.98)	13 (16.46)	20 (8.85)	59 (12.02)	

### 3.2 Integrative construction of a prognostic model

**Figure 2:**
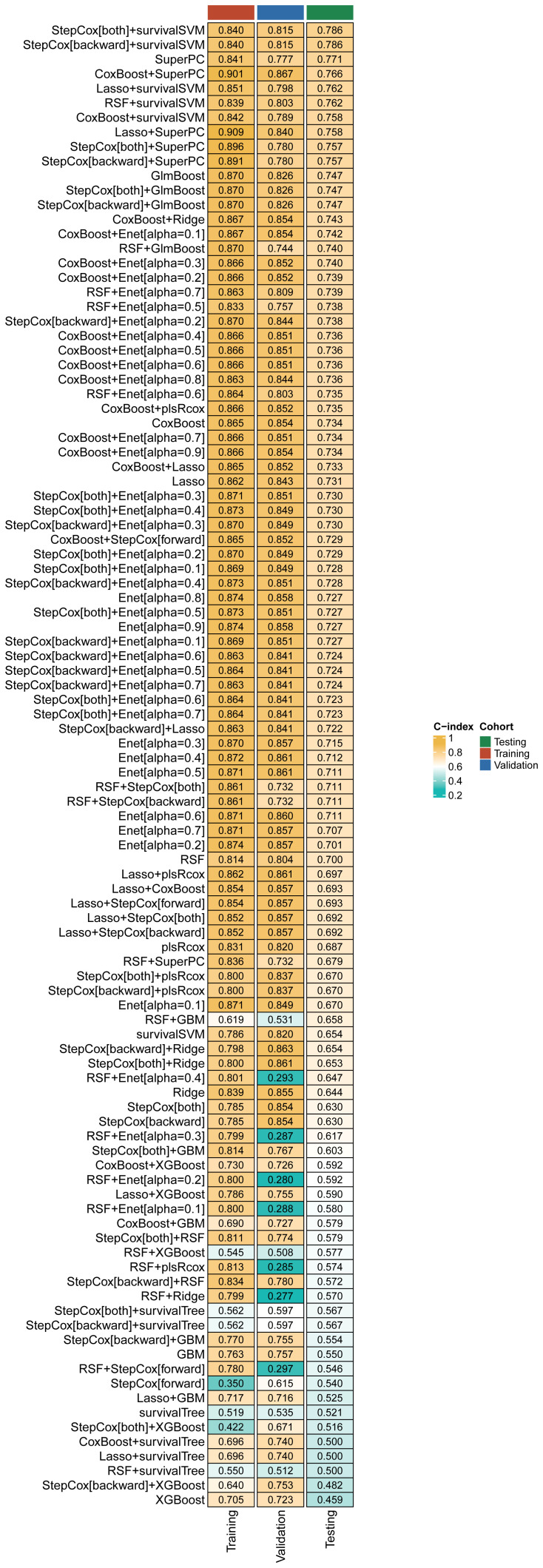
A comprehensive set of 119 prediction models and their predictive performance was systematically evaluated in the training, validation and testing cohorts using the C-index as the primary performance metric.

The relevant variables were used to conduct subsequent analyses. We conducted univariate Cox regression analysis on the training cohort and selected variables with p-values < 0.05 for the subsequent modeling process. **Supplementary Table S1** presents the variables along with their corresponding HR values associated with univariate Cox regression. After screening the 77 variables, 34 variables with p-values < 0.05 were identified, comprising 4 clinical baseline data, and 30 laboratory test results, which we utilized for modeling, however, no baseline medical history was incorporated. To this end, we combined 13 classical ML algorithms, including Least absolute shrinkage and selection operator (Lasso), CoxBoost, Supervised Principal Components (SuperPC), StepCox, Elastic network (Enet), survival Support Vector Machine (survival-SVM), Ridge, Partial least squares Regression for cox (plsRcox), Random Survival Forest (RSF), Gradient Boosting with Component wise Linear Models (GLMboost), Extreme Gradient Boosting (xgboost), survival tree, and Gradient Boosting Machine (GBM) for obtaining 119 algorithm combinations. A prognostic model requires a minimum of five variables to be considered valid (13). **Figure 2** depicts the combination algorithm for ML. The combination of stepcox[both] and survivalSVM algorithms demonstrated strong predictive capability, with a C-index of 0.840,0.815, and 0.786 in training, validation, and testing cohorts respectively.

### 3.3 Model comparison and assessment

**Figure 3:**
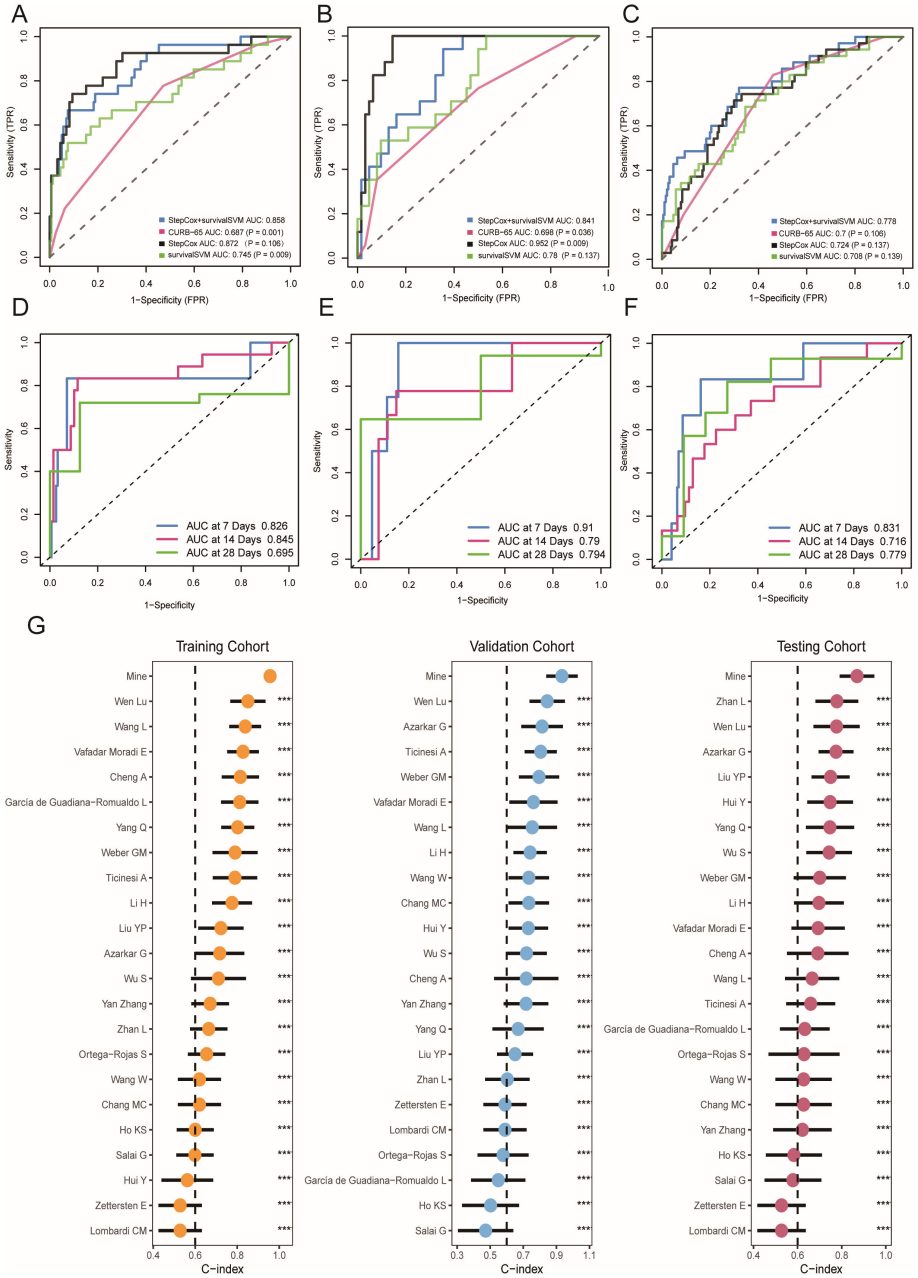
Prediction performance of ML-based model in training, validation and testing cohort. **(A–C)** Receiver operating characteristic (ROC) curve of ML-based model and other evaluation methods. **(D–F)** Time-dependent receiver operating characteristic (ROC) curve of ML-based model in 7-days, 14-days and 28-days. **(G)** Comparisons of ML-based model and other published models. *** p < 0.001.

To assess the predictive ability of our model, we compared its performance with traditional evaluation methods on the training, validation, and testing cohort using ROC curves. Our results showed that the AUC value of our model was 0.858 in the training cohort, whereas the AUC values of CURB-65, stepcox, and survivalSVM models were 0.687, 0.872, and 0.745 respectively (**Figure 3A**). The ROC curves of the training and validation cohorts were also plotted simultaneously. As depicted in **Figures 3B, C**, the AUC values of the combined ML model, CURB-65, stepcox, and survivalSVM models in the validation cohort were 0.841, 0.698, 0.952, and 0.780 respectively. At the same time, the AUC values of CURB-65, stepcox, and survivalSVM models in the testing cohort were 0.778, 0.70, 0.724, and 0.708 respectively. Moreover, we generated ROC curves for days 7, 14, and 28, showing excellent predictive performance of our model during these particular time intervals (**Figures 3D–F**). Crucially, our model exhibited stronger predictive capability relative to conventional methods. Moreover, we validated our model’s predictive performance by collecting relevant literature on COVID-19 prognostic models. To compare the performance of our model with previously published models, we collected relevant articles and parameters, as described in the Supplemental Material (**Supplementary Table S2**). In comparison, our model showed the best predictive performance in the training, validation, and testing cohorts, demonstrating its excellent usability (**Figure 3G**). Hence, all the results collectively demonstrate the reliability of our model.

### 3.4 Survival analysis and personalized clinical application

**Figure 4:**
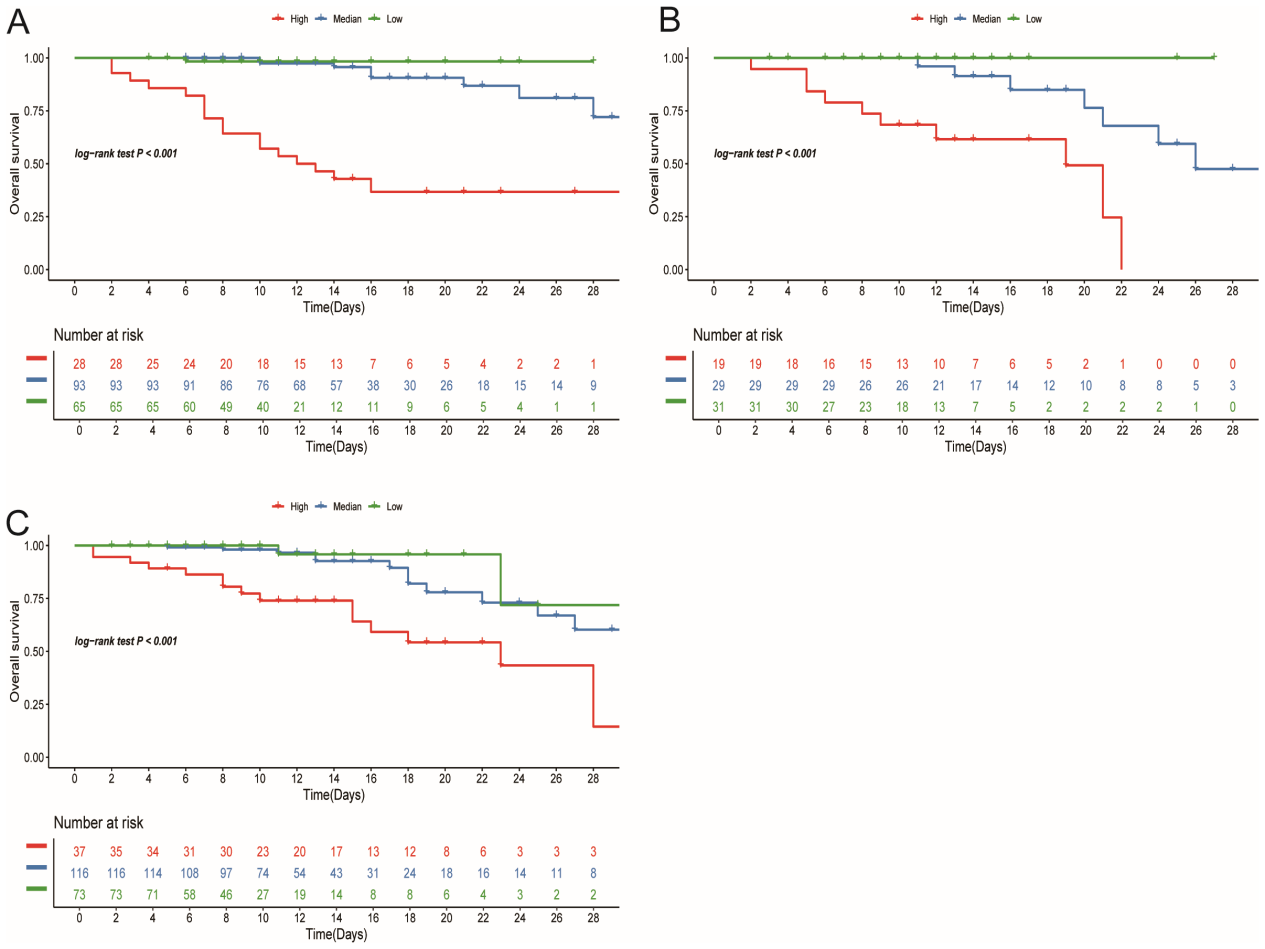
Stratification survival analysis in training **(A)**, validation **(B)**, and testing cohort **(C)**.

In the training cohort, patients were initially separated into low-, median-, and high-risk groups based on their risk scores. In this training cohort, the high-risk group represented 27 (15%) of patients, with the median-risk group containing 93 (50%) and the remaining 66 (35%) being categorized as low-risk. Setting these cutoff points allowed us to stratify patients and allocate clinical resources more rationally. Next, we plotted KM curves for each group based on the risk scores to visualize differences in survival rates. In the training cohort, the results indicated that there were significant differences in survival among 3 risk groups (P<0.05) (**Figure 4A**). To further verify the validity of our stratification scheme, we performed validation on the stratification indicators in both validation and testing cohorts (**Figures 4B, C**). Survival differences can be observed in all three cohorts (P<0.001). These findings solidify the reliability of our stratification scheme and provide a strong foundation for stratified treatment. According to the stratification group identified, it is recommended that patients in the low-risk group should be observed at home or followed up in a community hospital; patients in the median-risk group should be treated in a hospital with more specialized staff and equipment; patients in the high-risk group should be admitted to the intensive care unit (ICU) as soon as possible and given the necessary respiratory and circulatory support and symptomatic treatment.

### 3.5 Clinical and laboratory correlation analysis

**Figure 5:**
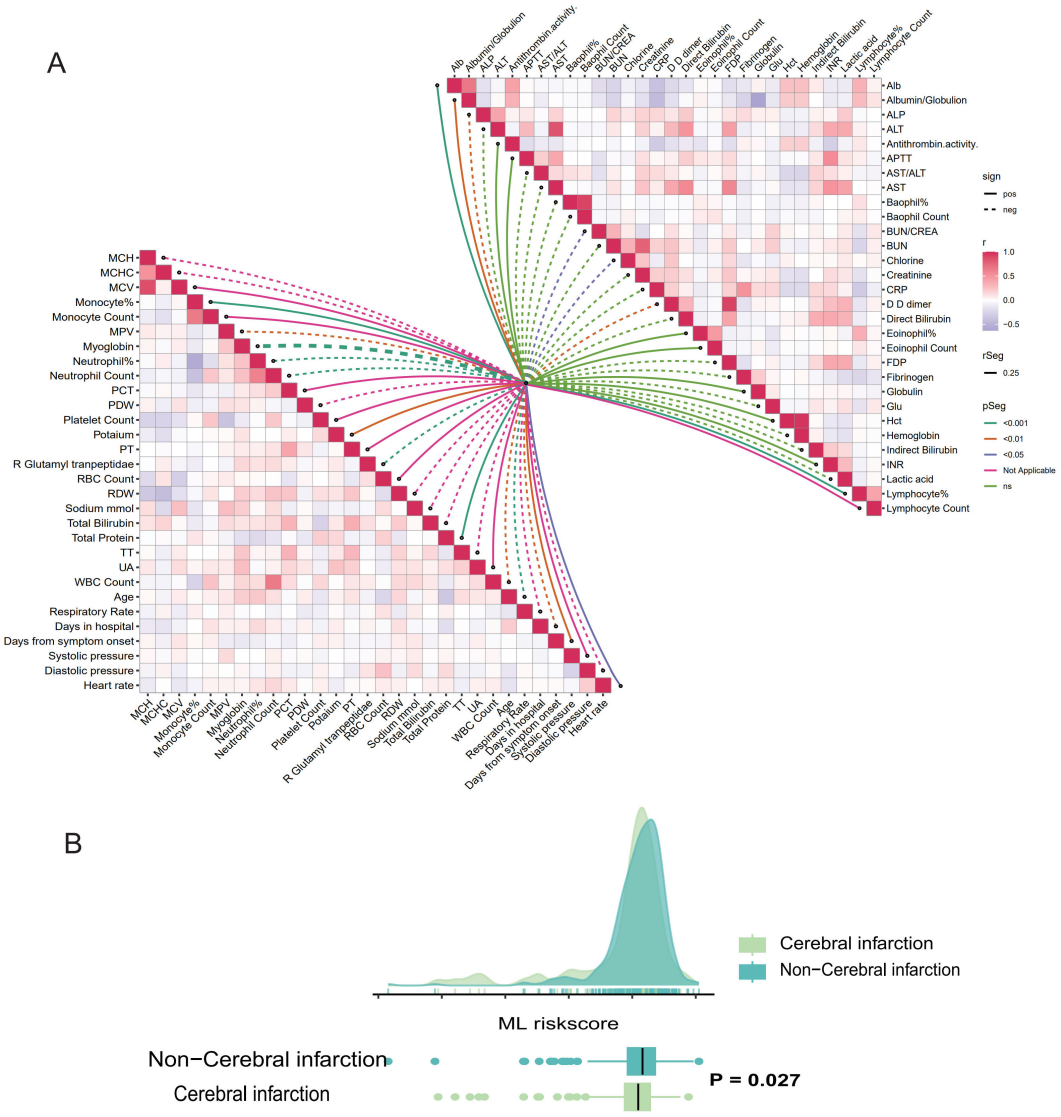
Correlation analysis between ML-based model and the results of clinical parameters and laboratory testing. **(A)** Butterfly plot demonstrates the correlation between the ML-based model and patients’ baseline characteristics, clinical vital signs, and laboratory results. **(B)** Raincloud plot illustrated the correlation between ML-model risk score and cerebral infarction.

In this study, a series of clinical baseline data and laboratory examination indicators of patients were collected. We used “ggcor” package to explore the correlation between risk score and patient baseline characteristics and laboratory examination indicators, as well as the correlation between various variables, and the results were presented in the form of a butterfly graph (**Figure 5A**). To explore the correlation between risk scores and underlying diseases, we compared the scores among patients, who had different underlying conditions. Analysis shows that patients with cerebral infarction have lower scores compared to those without these diseases, indicating a higher risk of death (**Figure 5B**). Supplementary Figure (**Supplementary Figures S1A–I**) elucidated the correlation between risk score and other underlying diseases.

## 4 Discussion

The winter of the outbreak of COVID-19 seems to be fading away, but the issues raised by this outbreak are worthy of deep consideration. The outbreak has not only led to a shortage of hospital beds and medical equipment but has also caused panic and concern among the population about whether they will be treated effectively. A simpler and more efficient risk assessment system is necessary to alleviate the shortage of medical resources and to better treat patients with COVID-19.

AI has been used extensively to predict the peak of epidemics in infected areas and to determine approximate outbreak control dates, among other things (21–23). In recent years, ML as an important part of AI has been effective in assessing disease severity, predicting patient prognosis, and providing early warning (24). In this study, due to the diversity of the data, we chose a combination of algorithms to construct the model. Compared to models from a single algorithm, The models constructed by the combined algorithm have higher stability, accuracy, diversity, and fewer instances of overfitting (10). By analyzing collected data from the blood tests and other medical history information of COVID-19 patients, we found that the prognostic model constructed using the ML combination of the stepcox[both]Lasso and survivalSVM algorithms exhibited strong predictive power. By comparison with other models, such as the CURB-65 and some are predictive models constructed by other researchers using ML learning (15–17, 25), we found the models constructed by this combination of stepcox[both] and survivalSVM algorithms have a higher C-index in the testing cohort, meaning that this model has stronger predictive performance.

In the model, blood test results included Albumin/Globulin, White blood cell (WBC) count, Neutrophil count, D-D dimer, supplement oxygen support, etc. These indicators are related to the degree of infection, nutritional status, and degree of damage to the heart, liver, and blood system. Some of the hematological indicators in this model are also consistent in the prognostic model of COVID-19 patients by Wen Lu et al. (26). Based on this predictive model, an early risk scoring system was developed to classify patients into low-, median-, and high-risk groups to facilitate a more rational allocation of healthcare resources. Then, it is recommended that COVID-19 patients be classified into 3 risk categories based on this risk scoring system and that patients in different subgroups be given different recommendations for medical care. This approach reduces avoidable initial admissions of low-risk patients and increases the attention of healthcare professionals to high-risk patients thereby reducing their risk of death. Since the algorithm used to construct the model was originally used for scoring the wear and tear of mechanical parts, a lower score for the patient in this model represents a higher level of risk. We also found that patients with cerebral infarction had significantly higher risk level in this study, which reminds us that patients with cerebral infarction infected with COVID-19 should be given increased attention and timely intervention. Research has shown that COVID-19 can cause a storm of inflammatory factors, a hypercoagulable state in the blood, and can also bind with angiotensin-converting enzyme 2 (ACE-2), which may lead to a series of problems such as hypertension and thrombosis (27). We speculate that patients with a history of cerebral infarction may experience further exacerbation of cerebrovascular disease and a higher risk of death after being infected with COVID-19.

Our prognostic model based on simple blood test indicators shows good predictive performance at all stages of the disease, and the combination of two ML algorithms makes it easier to simplify and transform (10). In addition, the derived early risk scoring system enables stratified management of COVID-19 patients, which is more suitable for the future clinical setting of PPPM. However, there are still several limitations to be addressed in this study. Firstly, the patient data we collected were all from elderly people in China, so the available sample size and the number of events for the outcomes of interest are limited, which may increase the risk of overfitting (9). Secondly, the prognosis of COVID-19 patients may vary in the medical context of different countries, so the early risk scoring system constructed may not apply to everyone. Although we have improved these limitations as much as possible by scientifically filtering the data and assigning training and validation sets, the usefulness of the system needs to be validated in a number of prospective multi center cohorts.

Overall, in an attempt to provide early individualized and scientific treatment recommendations for COVID-19 patients, a combination of ML algorithms was used to construct an optimal prognostic model based on biomarkers from blood tests. Moreover, the early risk scoring system constructed based on this model provided some references for predictive clinical care. Another winter could come at any time, but the lessons learned from COVID-19 will allow us to be well-prepared for similar difficulties.

## 5 Conclusion

In conclusion, we have developed a novel and highly accurate risk stratification method for COVID-19 that has a simple design and provides clinical decision support. The method has good clinical utility and can be readily implemented in diverse clinical settings.

## Data Availability

The raw data supporting the conclusions of this article will be made available by the authors, without undue reservation.
