# Supplementary material for: Development of a COVID-19 early risk assessment system based on multiple machine learning algorithms and routine blood tests: a real-world study
Source: Front Immunol. 2024 Sep 30;15:1430899. doi: 10.3389/fimmu.2024.1430899 (PMC11471604; doi:10.3389/fimmu.2024.1430899)
Supplement: Supplementary file 1 [file DataSheet1.docx]

Supplementary Material

# Supplementary Figures and Tables

## Supplementary Figures


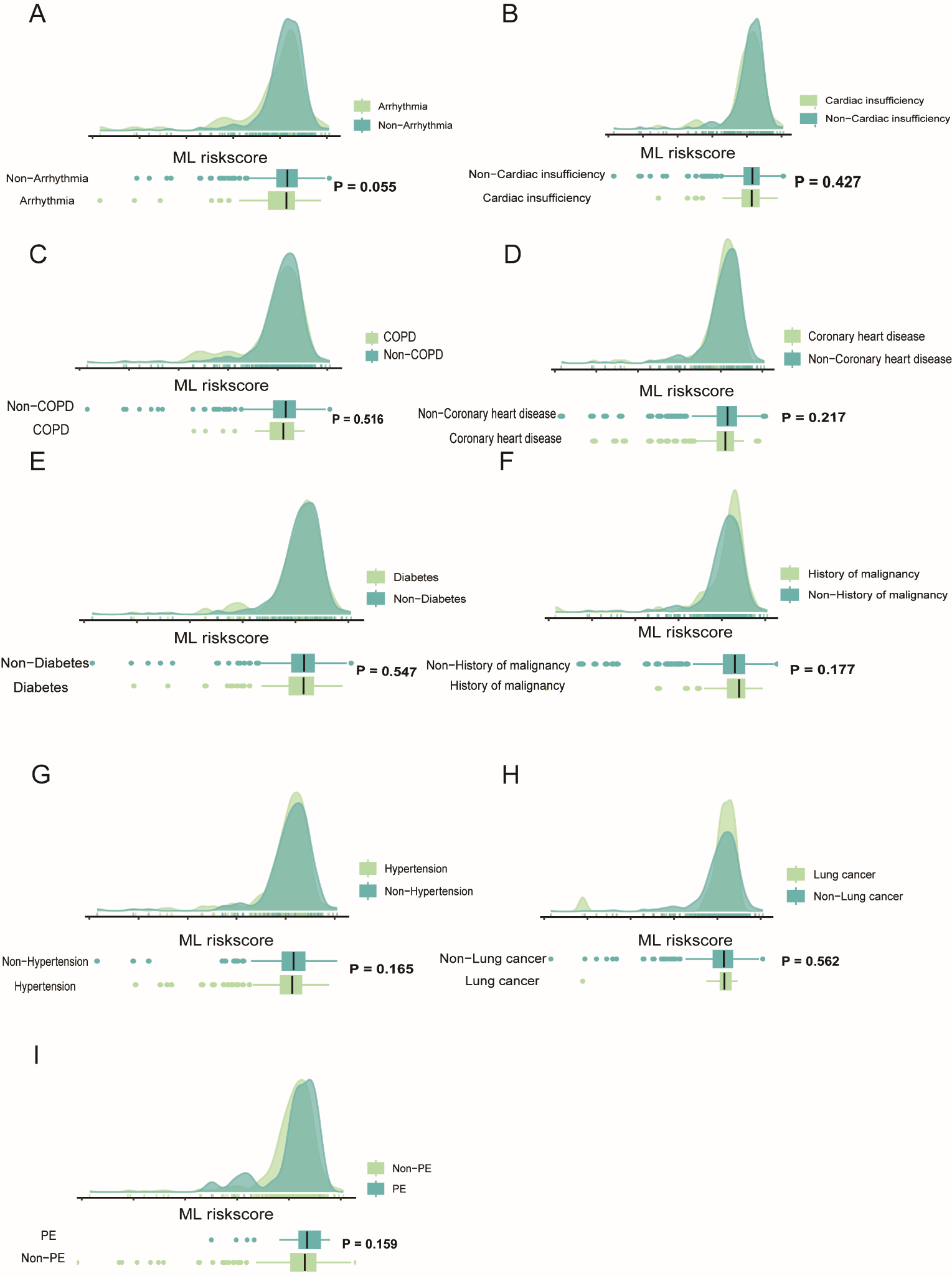


**Supplementary Figure 1.** Rainclouds of ML-model risk scores associated with arrhythmia, cardiac insufficiency, chronic obstructive pulmonary disease (COPD), coronary heart disease, diabetes, history of malignancy, hypertension, lung cancer, and pulmonary embolism (PE).

## Supplementary Tables

| Indicators | Training cohort | Validation cohort | Testing cohort | Total | P Value |
| --- | --- | --- | --- | --- | --- |
| Alb (median [IQR]) | 31.95 [29.40;35.00] | 32.10 [29.05;35.50] | 34.75 [31.60;37.90] | 33.10 [30.30;36.90] | 0 |
| Albumin/Globulin (mean (SD)) | 1.13 ± 0.22 | 1.11 (0.23) | 1.22 ± 0.23 | 1.17 ± 0.23 | 0 |
| ALP (median [IQR]) | 71.00 [60.00;89.00] | 73.00 [55.00;88.00] | 79.00 [64.00;98.00] | 75.00 [61.00;95.00] | 0.004 |
| ALT (median [IQR]) | 24.00 [17.00;39.00] | 25.00 [15.50;38.50] | 25.00 [16.00;41.00] | 25.00 [17.00;40.00] | 0.883 |
| Antithrombin activity (median [IQR]) | 78.00 [66.00;89.00] | 78.00 [65.50;89.50] | 91.00 [81.00;102.00] | 83.00 [71.50;96.00] | 0 |
| APTT (median [IQR]) | 29.35 [27.60;31.90] | 29.50 [26.55;32.70] | 30.35 [28.30;32.70] | 29.90 [27.70;32.50] | 0.085 |
| AST/ALT (median [IQR]) | 0.94 [0.71;1.43] | 1.08 [0.72;1.44] | 1.08 [0.77;1.50] | 1.04 [0.73;1.45] | 0.308 |
| AST (median [IQR]) | 25.00 [17.00;38.00] | 26.00 [18.00;34.00] | 27.00 [19.00;38.00] | 26.00 [18.00;37.50] | 0.29 |
| Basophil% (median [IQR]) | 0.10 [0.00;0.20] | 0.10 [0.00;0.20] | 0.20 [0.10;0.30] | 0.10 [0.10;0.30] | 0 |
| Basophil Count (median [IQR]) | 0.01 [0.00;0.01] | 0.01 [0.00;0.01] | 0.01 [0.01;0.03] | 0.01 [0.00;0.02] | 0 |
| BUN (median [IQR]) | 0.11 [0.08;0.13] | 7.00 [4.89;10.25] | 0.09 [0.07;0.12] | 0.10 [0.07;0.13] | 0.001 |
| Cardiac troponin I (median [IQR]) | 7.00 [5.32;9.55] | 0.01 [0.00;0.02] | 6.72 [5.23;8.92] | 6.93 [5.20;9.16] | 0.778 |
| Chlorine (median [IQR]) | 104.35[101.00;108.00] | 104.00 [101.40;107.15] | 105.25 [101.20;107.70] | 105.00 [101.05;107.80] | 0.7 |
| Creatinine (median [IQR]) | 64.10 [51.00;84.00] | 67.00 [55.00;92.25] | 69.00 [60.00;82.00] | 67.40 [55.20;85.00] | 0.06 |
| CRP (median [IQR]) | 28.00 [5.00;67.00] | 32.00 [11.50;97.50] | 28.87 [5.95;54.16] | 29.00 [6.00;57.93] | 0.016 |
| D-D dimer (median [IQR]) | 1.12 [0.60;2.65] | 1.12 [0.66;2.83] | 0.94 [0.54;1.78] | 1.03 [0.58;2.05] | 0.11 |
| Direct Bilirubin (median [IQR]) | 2.60 [1.60;3.60] | 2.90 [1.70;4.05] | 3.73 [2.72;5.59] | 3.10 [1.96;4.50] | 0 |
| Eosinophil% (median [IQR]) | 0.20 [0.10;0.90] | 0.30 [0.10;0.90] | 0.20 [0.10;0.30] | 0.20 [0.10;0.50] | 0.006 |
| Eosinophil Count (median [IQR]) | 0.00 [0.00;0.03] | 0.00 [0.00;0.02] | 0.01 [0.01;0.03] | 0.01 [0.00;0.02] | 0 |
| FDP (median [IQR]) | 3.45 [2.12;6.50] | 3.52 [2.17;6.66] | 5.00 [3.40;6.60] | 4.30 [2.80;6.60] | 0 |
| Fibrinogen (mean (SD)) | 3.94 ± 1.09 | 4.12 ±1.12 | 4.12 ± 0.99 | 4.05 ±1.05 | 0.193 |
| Globulin (median [IQR]) | 27.85 [25.30;31.30] | 28.70 [25.45;32.15] | 28.60 [26.20;32.00] | 28.40 [25.70;31.80] | 0.246 |
| Glu (median [IQR]) | 7.41 [5.72;10.10] | 7.50 [5.67;10.00] | 7.29 [5.97;10.00] | 7.40 [5.80;10.05] | 0.972 |
| Hct (median [IQR]) | 36.00 [33.30;39.00] | 35.50 [33.00;38.65] | 38.00 [34.20;41.70] | 36.60 [33.55;40.25] | 0 |
| Hemoglobin (median [IQR]) | 121.00[110.00;132.00] | 120.00 [110.00;131.00] | 127.00 [115.00;142.00] | 124.00 [112.00;137.00] | 0 |
| Indirect Bilirubin (median [IQR]) | 6.00 [4.50;8.10] | 5.90 [4.30;8.35] | 7.00 [5.52;8.78] | 6.50 [5.00;8.55] | 0.001 |
| INR (median [IQR]) | 1.03 [0.97;1.12] | 1.03 [0.97;1.12] | 1.02 [0.95;1.10] | 1.03 [0.96;1.11] | 0.232 |
| Lactic acid (median [IQR]) | 1.50 [0.00;2.10] | 1.21 [0.00;1.96] | 1.20 [0.80;2.00] | 1.30 [0.70;2.01] | 0.649 |
| Lymphocyte (median [IQR]) | 13.15 [8.30;21.10] | 13.10 [6.80;21.35] | 11.95 [6.70;18.70] | 12.60 [7.30;19.70] | 0.13 |
| Lymphocyte Count (median [IQR]) | 0.90 [0.63;1.43] | 0.85 [0.52;1.25] | 0.80 [0.60;1.20] | 0.85 [0.60;1.30] | 0.099 |
| MCH (median [IQR]) | 30.60 [29.40;31.70] | 30.90 [29.70;32.10] | 30.80 [29.60;32.10] | 30.70 [29.60;31.90] | 0.238 |
| MCHC (median [IQR]) | 337.00[329.00;343.00] | 339.00 [334.00;346.50] | 337.00 [329.00;345.00] | 337.00 [330.00;344.00] | 0.146 |
| MCV (median [IQR]) | 90.90 [87.50;94.50] | 91.40 [88.45;94.00] | 91.10 [88.10;94.50] | 91.10 [88.10;94.50] | 0.8 |
| Monocyte (median [IQR]) | 6.60 [4.70;8.80] | 7.10 [4.35;8.95] | 6.50 [3.90;8.40] | 6.60 [4.20;8.80] | 0.282 |
| Monocyte Count (median [IQR]) | 0.48 [0.32;0.67] | 0.43 [0.30;0.56] | 0.50 [0.30;0.60] | 0.47 [0.30;0.61] | 0.382 |
| MPV (median [IQR]) | 9.50 [8.70;10.30] | 9.60 [8.80;10.36] | 10.10 [9.30;10.70] | 9.80 [9.00;10.60] | 0 |
| Myoglobin (median [IQR]) | 44.10 [23.60;109.50] | 53.60 [31.70;98.95] | 29.15 [18.70;61.50] | 37.80 [22.10;78.25] | 0 |
| Neutrophil% (median [IQR]) | 78.90 [68.50;85.30] | 77.90 [67.95;87.85] | 78.75 [70.90;88.70] | 78.60 [69.70;86.75] | 0.13 |
| Neutrophil Count (median [IQR]) | 5.33 [3.50;7.82] | 4.88 [3.61;7.49] | 5.66 [3.95;8.25] | 5.53 [3.75;7.96] | 0.255 |
| PCT (median [IQR]) | 0.06 [0.04;0.14] | 0.06 [0.04;0.14] | 0.10 [0.05;0.55] | 0.07 [0.04;0.24] | 0 |
| PDW (median [IQR]) | 16.30 [16.00;16.70] | 16.40 [16.00;16.90] | 13.50 [10.80;16.10] | 16.10 [13.00;16.50] | 0 |
| Platelet Count (median [IQR]) | 223.50[162.00;283.00] | 197.00 [159.00;244.50] | 198.00 [142.00;264.00] | 206.00 [151.00;267.00] | 0.067 |
| Potassium (median [IQR]) | 3.62 [3.39;4.05] | 3.70 [3.42;4.00] | 3.76 [3.40;4.12] | 3.70 [3.40;4.08] | 0.513 |
| PT (median [IQR]) | 11.90 [11.20;12.90] | 11.90 [11.20;12.90] | 11.65 [10.90;12.40] | 11.80 [11.00;12.70] | 0.024 |
| R Glutamyl transpeptidase (median [IQR]) | 34.00 [21.00;58.00] | 37.00 [24.00;59.50] | 42.00 [25.00;81.00] | 38.00 [23.00;70.50] | 0.029 |
| RBC Count (median [IQR]) | 3.92 [3.60;4.34] | 3.97 [3.53;4.23] | 4.19 [3.76;4.54] | 4.04 [3.65;4.44] | 0 |
| RDW (median [IQR]) | 13.20 [12.60;13.80] | 13.20 [12.50;13.90] | 13.20 [12.50;14.50] | 13.20 [12.55;14.10] | 0.765 |
| Sodium (median [IQR]) | 138.00[134.00;140.80] | 137.00 [134.75;140.35] | 140.35 [137.30;143.10] | 139.00 [135.90;142.00] | 0 |
| Total Bilirubin (median [IQR]) | 8.80 [6.50;12.10] | 9.10 [6.95;12.15] | 11.25 [8.61;14.26] | 9.70 [7.51;13.30] | 0 |
| Total Protein (median [IQR]) | 60.25 [56.10;64.60] | 61.90 [56.75;65.60] | 64.15 [60.00;68.30] | 62.40 [57.35;66.85] | 0 |
| TT (median [IQR]) | 13.95 [13.10;15.20] | 13.90 [12.95;15.60] | 13.90 [13.00;15.10] | 13.90 [13.10;15.10] | 0.892 |
| UA (median [IQR]) | 260.00[211.00;329.00] | 275.40 [218.50;339.50] | 251.00 [195.00;314.00] | 260.00 [207.00;323.00] | 0.063 |
| WBC Count (median [IQR]) | 7.28 [5.15;9.74] | 6.34 [5.04;8.80] | 7.40 [5.50;9.80] | 7.20 [5.34;9.59] | 0.198 |

**Supplementary Table 1.** Laboratory blood results from patients in training, validation, and testing cohorts.

| PMID | Model | Symbol |
| --- | --- | --- |
| 32446795 | Yan Zhang | Diabetes |
| 32446795 | Yan Zhang | Glu |
| 32556293 | Wu S | Age score |
| 32556293 | Wu S | CRP |
| 32556293 | Wu S | Lymphocyte Count |
| 32556293 | Wu S | Lymphocyte Count |
| 32556293 | Wu S | PCT |
| 32566572 | Liu YP | CRP |
| 32566572 | Liu YP | NLR |
| 32631365 | Yang Q | D-D dimer |
| 32631365 | Yang Q | hypertension |
| 32631365 | Yang Q | NLR |
| 32712332 | Cheng A | BUN |
| 32712332 | Cheng A | D-D dimer |
| 32842719 | Chang MC | Glu |
| 33333477 | Vafadar Moradi E | Age score |
| 33333477 | Vafadar Moradi E | NLR |
| 33333477 | Vafadar Moradi E | WBC Count |
| 33382747 | Hui Y | Age score |
| 33382747 | Hui Y | Diabetes |
| 33382747 | Hui Y | Lymphocyte Count |
| 33632273 | Zettersten E | gender |
| 33647451 | Ho KS | asthma |
| 33647451 | Ho KS | Eosinophil Count |
| 33762058 | Wen Lu | Age score |
| 33762058 | Wen Lu | BUN |
| 33762058 | Wen Lu | CRP |
| 33762058 | Wen Lu | Lymphocyte Count |
| 33762058 | Wen Lu | Lymphocyte Count |
| 33762058 | Wen Lu | Platelet Count |
| 33762058 | Wen Lu | WBC Count |
| 33879073 | Wang L | Age score |
| 33879073 | Wang L | WBC Count |
| 34272635 | García de Guadiana-Romualdo L | D-D dimer |
| 34289910 | Azarkar G | comorbidity |
| 34289910 | Azarkar G | CRP |
| 34289910 | Azarkar G | hypertension |
| 34289910 | Azarkar G | Lymphocyte Count |
| 34289910 | Azarkar G | Platelet Count |
| 34326704 | Ticinesi A | PCT |
| 34335053 | Zhan L | NLR |
| 34728168 | Aguirre-García GM | Age score |
| 34728168 | Aguirre-García GM | comorbidity |
| 34728168 | Aguirre-García GM | Diabetes |
| 34728168 | Aguirre-García GM | hypertension |
| 34728168 | Aguirre-García GM | roomair |
| 34728168 | Aguirre-García GM | eGFR |
| 34956098 | Wang W | Glu |
| 35287158 | Lombardi CM | gender |
| 35697747 | Weber GM | Age score |
| 35697747 | Weber GM | Alb |
| 35697747 | Weber GM | AST |
| 35697747 | Weber GM | Creatinine |
| 35697747 | Weber GM | CRP |
| 35697747 | Weber GM | WBC Count |
| 35855759 | Li H | D-D dimer |
| 35855759 | Li H | Glu |
| 35937941 | Ortega-Rojas S | NLR |
| 35937941 | Ortega-Rojas S | PLR |
| 37093279 | Salai G | Eosinophil Count |
|  | ML model | Albumin/Globulin |
|  | ML model | AST/ALT |
|  | ML model | AST |
|  | ML model | Basophil |
|  | ML model | Chlorine |
|  | ML model | CRP |
|  | ML model | D-D dimer |
|  | ML model | Direct Bilirubin |
|  | ML model | FDP |
|  | ML model | Monocyte |
|  | ML model | Myoglobin |
|  | ML model | Neutrophil |
|  | ML model | Neutrophil Count |
|  | ML model | PCT |
|  | ML model | PT |
|  | ML model | RDW |
|  | ML model | Total Bilirubin |
|  | ML model | WBC Count |
|  | ML model | Supplement oxygen support |
|  | ML model | Age |

**Supplementary Table 2.** Variables that were published in COVID-19 related literature previously and in our ML model.
